# Prevalence of type 2 diabetes and pre‐diabetes among pulmonary and extrapulmonary tuberculosis patients of Bangladesh: A cross‐sectional study

**DOI:** 10.1002/edm2.334

**Published:** 2022-03-08

**Authors:** Afsana Habib Sheuly, S. M. Zahid Hassan Arefin, Lingkan Barua, Muhammed Shahriar Zaman, Hasina Akhter Chowdhury

**Affiliations:** ^1^ Helen Keller International Bangladesh Dhaka Bangladesh; ^2^ Department of Biostatistics Bangladesh University of Health Sciences (BUHS) Dhaka Bangladesh; ^3^ Department of Epidemiology and Biostatistics Institute of child and Mother Health Dhaka Bangladesh; ^4^ Department of Noncommunicable Diseases Bangladesh University of Health Sciences (BUHS) Dhaka Bangladesh; ^5^ School of Rehabilitation Therapy Queen’s University Kingston Ontario Canada; ^6^ 535595 Centre for Injury Prevention and Research Bangladesh Dhaka Bangladesh

**Keywords:** pre‐diabetes, tuberculosis, type 2 diabetes

## Abstract

**Background:**

We aimed to determine the prevalence of type 2 diabetes (T2D) and pre‐diabetes (pre‐DM) among patients with pulmonary tuberculosis (PTB) and extrapulmonary tuberculosis (EPTB) in Bangladesh. We also examined the association between type of TB and hyperglycaemia as an adjunct to the primary objective.

**Materials and Methods:**

This cross‐sectional analytical study recruited 350 TB patients (175 PTB and 175 EPTB) from two tertiary care hospitals specialized for TB treatment. Oral glucose tolerance tests and fasting plasma glucose measurements were carried out for unknown glycaemic status and those with previously known diabetes, respectively.

**Results:**

Overall, the prevalence of T2D and pre‐DM was 19.1% (new 85.1%, old 14.9%) and 34.3%, respectively. Although the risk factors were highly prevalent among the patients with EPTB, a higher proportion of T2D (26.3%) and pre‐DM (34.3%) was detected among the patients with PTB. The proportion of impaired fasting glucose was low in both groups, but a high trend of impaired glucose tolerance was observed across the groups, with a higher proportion (35.4%) in the PTB group. Both pre‐DM and T2D showed significantly higher odds (pre‐DM, AOR: 4.488; CI: 2.531–7.958; *p* < .001 and T2D, AOR: 4.280; CI: 2.305–7.946; *p* < .001) for having PTB.

**Conclusion:**

The prevalence of T2D and pre‐DM was higher among the patients with PTB, and it (PTB) appeared as a predictor of hyperglycaemia. It indicates the primary intervention should target the patients with PTB to get the maximum benefit of screening to reduce the number of risk factors, disease burden and subsequent complications.

## Graphical abstract

First comparative cross‐sectional analytical study of Bangladesh that found a high prevalence of T2D and pre‐DM among the patients with pulmonary tuberculosis. Again, pulmonary TB appeared as a significant predictor of T2D and pre‐DM among the study population.

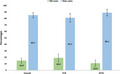

## 1 INTRODUCTION

Bangladesh is a lower‐middle‐income country facing a dual burden of communicable and noncommunicable diseases (NCDs).^1^ Among the communicable diseases, tuberculosis (TB) is considered a public health problem, and Bangladesh enlisted as one of the 30 high TB burden countries of the world.^2^ In Bangladesh, the incidence and prevalence of all forms of TB (pulmonary and extrapulmonary) in 2015 were 225 and 382 per 100,000 population, respectively.^3^ On the other hand, the mortality rate was 45 per 100000 people during the aforementioned year.^3^ Besides, the country is also on the list of 30 high multidrug‐resistant tuberculosis (MDR‐TB) burden countries in the world.^2^

Among the NCDs, diabetes mellitus (DM) has ranked the 7th leading cause of global death in 2016,^4^ and its current global prevalence is 9.3% in 2019.^5^ Although previously it was considered a disease of the affluent society, now it is a public health problem in low‐ and middle‐income countries^6^ like Bangladesh. As per the epidemiology of type 2 diabetes (T2D), it is six times more common in people of South Asian descent compared to other ethnic groups.^7^ A recent national survey reported the prevalence of raised blood glucose is 8.3% in Bangladesh.^1^ Previous studies informed the increasing trend of T2D in Bangladesh.^8^, ^9^, ^10^ Such an increased trend of DM simultaneously increases the burden of TB among the general population.^11^

Previous studies have shown that the relation of DM and TB acts as a two‐edged sword.^12^, ^13^, ^14^ Because DM increases the risk of TB, patients with TB have higher rates of DM.^12^, ^13^, ^14^ Again, TB can even cause diabetes in those not previously known to be diabetic.^15^ Thus, TB and DM are a potentially lethal combination having a synergistic effect and is also a growing public health challenge for developing countries where both diseases are most likely to converge.^16^ As a result, the health system needs to coordinate and ensure continuity of the long‐term care required for DM with the immediate short‐term required for TB control.^17^ However, globally data related to T2D among TB patients are scanty. More specifically, very few studies reported a clear comparison of T2D among pulmonary tuberculosis (PTB) and extrapulmonary tuberculosis (EPTB) patients. In this regard, most recent global evidence reported the pooled prevalence of diabetes was 15·3% among patients with tuberculosis.^18^ Although the coexistence of these two diseases is very common in developing countries with lower socioeconomic status, rarely do they try to explore the situation. In Bangladesh, we have not yet found any study that reported the distribution of diabetes and pre‐diabetes among the PTB and EPTB patients to elucidate a real scenario that will guide the efforts to halt both epidemics. Hence, in this study, we tried to evaluate the prevalence of pre‐diabetes and diabetes among patients with PTB and EPTB. As an adjunct, we also examined the association between type of TB and hyperglycaemia (pre‐DM, T2D) to identify the future target group of intervention.

## 2 MATERIALS AND METHODS

### 2.1 Study design and settings

This was a cross‐sectional analytical study conducted in two tertiary care hospitals that are specialized in tuberculosis treatment: one was the National Institute of Diseases of the Chest and Hospital (NIDCH) and another was National Centre for Tuberculosis & Research (NCTBR).

### 2.2 Recruitment of study population

A total of 350 TB patients (175 PTB and 175 EPTB) were recruited purposively from all consecutive patients who fulfilled the inclusion criteria of the study as follows: (i) both pulmonary and extrapulmonary TB cases diagnosed based on national guideline,^19^ (ii) patients with multidrug‐resistant TB, (iii) age 18 years and above, (iv) both men and women, (v) physically able and willing to participate. Again, exclusion criteria were as follows: (i) patients who had chronic illnesses such as cancer, HIV/AIDS (ii) who were mentally challenged or institutionalized in a hospital, prison and nursing home. The detailed sample size calculation was added as supporting information (Table S1). These patients underwent treatment at inpatient departments as well as at DOTs centres of the concerned hospitals during the study period (10 January 2015 to 31 December 2015).

### 2.3 Data collection procedures

Data were collected using a pre‐tested interviewer‐administered semi‐structured questionnaire filled out in three phases: asking questions, physical measurement and blood glucose measurement.

#### 2.3.1 First phase (asking questions)

A face‐to‐face interview collected information (personal, sociodemographic, co‐morbidities/complications, family history, risk factors, TB treatment categories, diabetes‐related information) using the questionnaire.

#### 2.3.2 Second phase (physical measurement)

In this phase, anthropometric values (height, weight and waist circumference) and blood pressure (BP) were measured by trained male and female assistants with maintaining adequate privacy. Bodyweight in light cloths was measured using a Soehenle mechanical weighing scale (Soehenle‐Waagen GmbH & Co.KG, Wilhelm‐Soehenle‐Strabe 2, D‐71540 Murrhard/Germany), and the height was measured using a steel tape. Body Mass Index (BMI) was calculated as the ratio of weight in kilograms over height in metres squared [weight/height (kg/m²)]. For waist circumference, a tape was placed horizontally midway between the two points (the lowest rib margin and the iliac crest in the mid‐axillary line) to measure the circumference. Two blood pressure readings were recorded using a standard sphygmomanometer (Model UA‐767, A & D Company Limited, Tokyo, Japan). The mean of the two measurements determined the final value of blood pressure.

#### 2.3.3 Third phase (blood glucose measurement)

In this final phase, to measure blood glucose, 5 ml of venous blood was collected with aseptic precaution at fasting state, and again 3cc was collected after two hours (2‐h) of 75 g glucose intake. Oral glucose tolerance tests and fasting plasma glucose measurements were carried out for unknown glycaemic status and those with previously known diabetes, respectively. We measured the fasting and 2‐h plasma glucose on the same day. We used the laboratory‐accredited Glucose Oxidase method (Randox Laboratories Ltd., UK).

### 2.4 Ascertainment of key variables

#### 2.4.1 Pulmonary TB

A patient with

1. at least one sputum specimen positive for acid‐fast bacilli (AFB) including any scanty result or
2. symptoms suggestive of TB with two sputum specimens negative for AFB and positive on Xpert MTB/RIF test or
3. above two options were negative but chest X‐ray abnormalities consistent with active TB and diagnosed by qualified physician.^19^

#### 2.4.2 Extrapulmonary TB

A patient with TB of organs other than the lungs as confirmed by a qualified physician.^19^

#### 2.4.3 Category I TB treatment

Category I was for new smear‐positive patients/new smear‐negative patients with pulmonary TB with extensive parenchymal involvement, having concomitant HIV or severe form of EPTB.^19^

#### 2.4.4 Category II TB treatment

Category II was for sputum smear‐positive patients who have relapsed, who have treatment failure or who are receiving treatment after treatment interruption.^19^

#### 2.4.5 Category III TB treatment

Category III was for new smear‐negative pulmonary TB patients (other than those in Category 1), and patients with new less severe forms of extrapulmonary TB.^20^

#### 2.4.6 Pre‐diabetes mellitus (Pre‐DM)

Presence of impaired glucose tolerance (IGT) and/or impaired fasting glucose (IFG) was defined as pre‐DM. Here, the cut‐off for IGT was fasting plasma glucose <7.0 mmol/l (126 mg/dl) and 2–h plasma glucose ≥7.8 and <11.1mmol/l (140 mg/dl and 200 mg/dl). Again, IFG was defined as fasting plasma glucose 6.1 to 6.9 mmol/l (110mg/dl to 125mg/dl) and 2–h plasma glucose <7.8 mmol/l (140 mg/dl).^21^

#### 2.4.7 Diabetes mellitus (DM)

We diagnosed diabetes using WHO criteria as follows: fasting plasma glucose ≥7.0 mmol/L (126 mg/dl) or 2‐h plasma glucose ≥11.1 mmol/L (200 mg/dl) and self‐statement of a person as known diabetic or on anti‐diabetic medication.^21^ Here, new cases were those diagnosed for the first time in the study, and old cases were those previously diagnosed by a health professional and/or documented anti‐diabetic drug intake for the raised blood glucose.

#### 2.4.8 Generalized obesity

The participants were considered obese and overweight based on the WHO classification for Asian population (BMI ≥27.5 kg/m^2^, obesity; BMI 23.0–27.4 kg/m^2^, overweight).^22^

#### 2.4.9 Central obesity

Central obesity was categorized according to the cut‐off value specified by the International Diabetes Federation—waist–circumference >94 centimetre for men and >80 centimetre for women.^23^

#### 2.4.10 Hypertension

When systolic BP ≥140 mm Hg and/or diastolic BP ≥90 mm Hg and/or on antihypertensive treatment for raised BP.^24^

### 2.5 Statistical analysis

We used the software Statistical Product and Service Solutions 20.0 for Windows (SPSS, Inc., Chicago, IL, USA). All the estimates of precision were presented at a 95% confidence interval (CI) as appropriate. Descriptive analysis included mean, standard deviation (SD), median and interquartile range (IQR), frequencies and percentages.

The association between the type of tuberculosis (PTB, EPTB) and the high blood glucose level (T2D, pre‐DM) was evaluated using multinomial logistic regression analysis. To run multinomial logistic regression, we checked the assumptions: independence of observations, multicollinearity and outliers. We did not find any violation of these assumptions. Then, we run a univariate analysis between blood glucose levels and the type of TB to evaluate the initial finding of association to predict the impact of an independent variable on the dependent variable in the subsequent regression. We found a highly significant association (*p* < .001) between blood glucose levels and the type of TB that justified running further regression analysis to generate an odds ratio (OR). The statistical tests were considered significant (2‐sided) at a level of *p* < .05.

## 3 RESULT

### 3.1 Background profile of the study participants

The overall mean age of the participants was 36.3 ± 12.1 years, without a statistically significant mean age difference (*p* = .74) between the groups (Table 1). Most of the participants were male (67.7%), literate (93.7%) and service holder (25.4%). The majority of the participants (66.9) came from lower‐middle‐income groups and residing in the Dhaka division (64%). Group‐wise distribution showed the mean income of the PTB group (BDT 15,097 ± 11,261) was significantly lower (*p* < .0001) than the mean income of the EPTB group (BDT 20,629 ± 11,696). PTB is more common among the housewives (24%) and residents living outside the Dhaka division (68%). Most of the participants with PTB were under category 3 treatment (49.7%), while most of the participants with EPTB were under category 1 (94.9%).

**TABLE 1:** Background profile of the study participants by category of tuberculosis, *n* = 350

Variables	Pulmonary TB (*n* = 175)		Extrapulmonary TB (*n* = 175)		Total (*n* = 350)	
	*n* (%)	95% CI	*n* (%)	95% CI	*n* (%)	95% CI
Age (years)^a^	36.8 ± 13		35.8 ± 11.1		36.3 ± 12.1	
Age categories (years)						
≤25	39 (22.3)	16.1–28.5	36 (20.6)	14.6–26.6	75 (21.4)	17.1–25.7
26–35	61 (34.9)	27.8–42	63 (36)	28.9–43.1	124 (35.4)	30.4–40.4
36–45	30 (17.1)	11.5–22.7	40 (22.9)	16.7–29.1	70 (20)	15.8–24.2
>45	45 (25.7)	19.2–32.2	36 (20.6)	14.6–26.6	81 (23.1)	18.7–27.5
Sex						
Men	117 (66.9)	59.9–73.9	120 (68.6)	61.7–75.5	237 (67.7)	62.8–72.6
Women	58 (33.1)	26.1–40.1	55 (31.4)	24.5–38.3	113 (32.3)	27.4–32.2
Living area						
Dhaka division	56 (32)	25.1–38.9	168 (96)	93.1–98.9	224 (64)	59–69
Other division	119 (68)	61.1–74.9	7 (4)	1.1–6.9	126 (36)	31–41
Educational status						
Illiterate	21 (12.0)	7.2–16.8	1 (0.6)	−0.5 to 1.7	22 (6.3)	3.8–8.8
Literate	154 (88)	83.2–92.8	174 (99.4)	98.3–100.5	328 (93.7)	91.2–96.2
Occupational status						
Service	33 (18.9)	13.1–24.7	56 (32)	25.1–38.9	89 (25.4)	20.8–30
Business	34 (19.4)	13.5–25.3	49 (28)	21.3–34.7	83 (23.7)	19.2–28.2
Housewife	42 (24)	17.7–30.3	30 (17.1)	11.5–22.7	72 (20.6)	16.4–24.8
Day labourer	25 (14.3)	9.1–19.5	17 (9.7)	5.3–14.1	42 (12)	8.6–15.4
Others	41 (23.4)	17.1–29.7	23 (13.1)	8.1–18.1	64 (18.3)	14.2–22.4
Monthly household income (BDT)^b^	12,000 (9000–20,000)		20,000 (11,000–30,000)		15,000 (10,000–20,000)	
Monthly income groups^c^						
≤7232 (low)	33 (18.9)	13.1–24.7	15 (8.6)	4.4–12.8	48 (13.7)	10.1–17.3
7233–28,189 (lower‐middle)	123 (70.3)	63.5–77.1	111 (63.4)	56.3–70.5	234 (66.9)	62–71.8
≥28,190 (upper‐middle and above)	19 (10.9)	6.3–15.5	49 (28)	21.3–34.7	68 (19.4)	15.3–23.5
Family history of TB	50 (28.6)	21.9–35.3	42 (24)	17.7–30.3	92 (26.3)	21.7–30.9
Treatment categories						
Category 1	75 (42.9)	35.6–50.2	166 (94.9)	91.6–98.2	241 (68.9)	64.1–73.7
Category 2	13 (7.4)	3.5–11.3	3 (1.7)	−0.2 to 3.6	16 (4.6)	2.4–6.8
Category 3	87 (49.7)	42.3–57.1	6 (3.4)	0.7–6.1	93 (26.6)	22–31.2
Family history of diabetes	40 (22.9)	16.7–29.1	44 (25.1)	18.7–31.5	84 (24)	19.5–28.5
Current tobacco user^d^	85 (48.6)	41–56.2	61 (34.9)	27.8–42.4	146 (41.7)	36.5–47.1
Body‐mass‐index^a^, ^e^	17.8 (3.3)	20 (3)	18.9 (3.3)			
Waist circumference^a^, ^e^	73.0 (8.3)	83.2 (9.8)	78.1 (10.4)			
Overweight^d^	6 (3.4)	0.7–6.1	23 (13.1)	8.1–18.1	29 (8.3)	5.4–11.2
Generalized obesity^d^	3 (1.7)	−0.2 to 3.6	9 (5.1)	1.8–8.4	12 (3.4)	1.5–5.3
Central obesity^d^	14 (8)	4–13	34 (19.4)	13.8–26.1	48 (13.7)	10.3–17.8
Hypertension^d^	7 (4)	1.6–8.1	18 (10.3)	6.2–158	25 (7.1)	4–10.4

Note

All the variables are presented using frequency and percentages unless otherwise indicated using uniform requirements for manuscripts submitted to biomedical journals.

Abbreviation: TB, tuberculosis.

^a^ Representing mean and standard deviation.

^b^ Presented as median with interquartile range.

^c^ According to the July 2019 per‐capita gross national income (GNI) and the World Bank calculation.

^d^ Statistically significant *p* < .05 based on Chi‐square test.

^e^ Statistically significant *p* < .05 based on Mann–Whitney *U* test.

Overall, 24% of the participants had a family history of DM. The number of tobacco users was significantly higher (*p* = .01) in the PTB group (41.7%). There were more participants (*p* = .03) in the EPTB group (10.3%) who were suffering from hypertension than in the PTB groups (4%). The mean body mass index (20 ± 3) and waist circumference (83.2 ± 9.8) were also significantly higher (*p* = .0001) among the EPTB group. The overall proportion of overweight participants was 8.3%. The trend of overweight was slightly higher in the EPTB group (13.1%) than that of the PTB group (3.4%), but the difference was statically significant (*p* = .001).

### 3.2 Type 2 diabetes and pre‐DM among the TB patients

**FIGURE 1:**
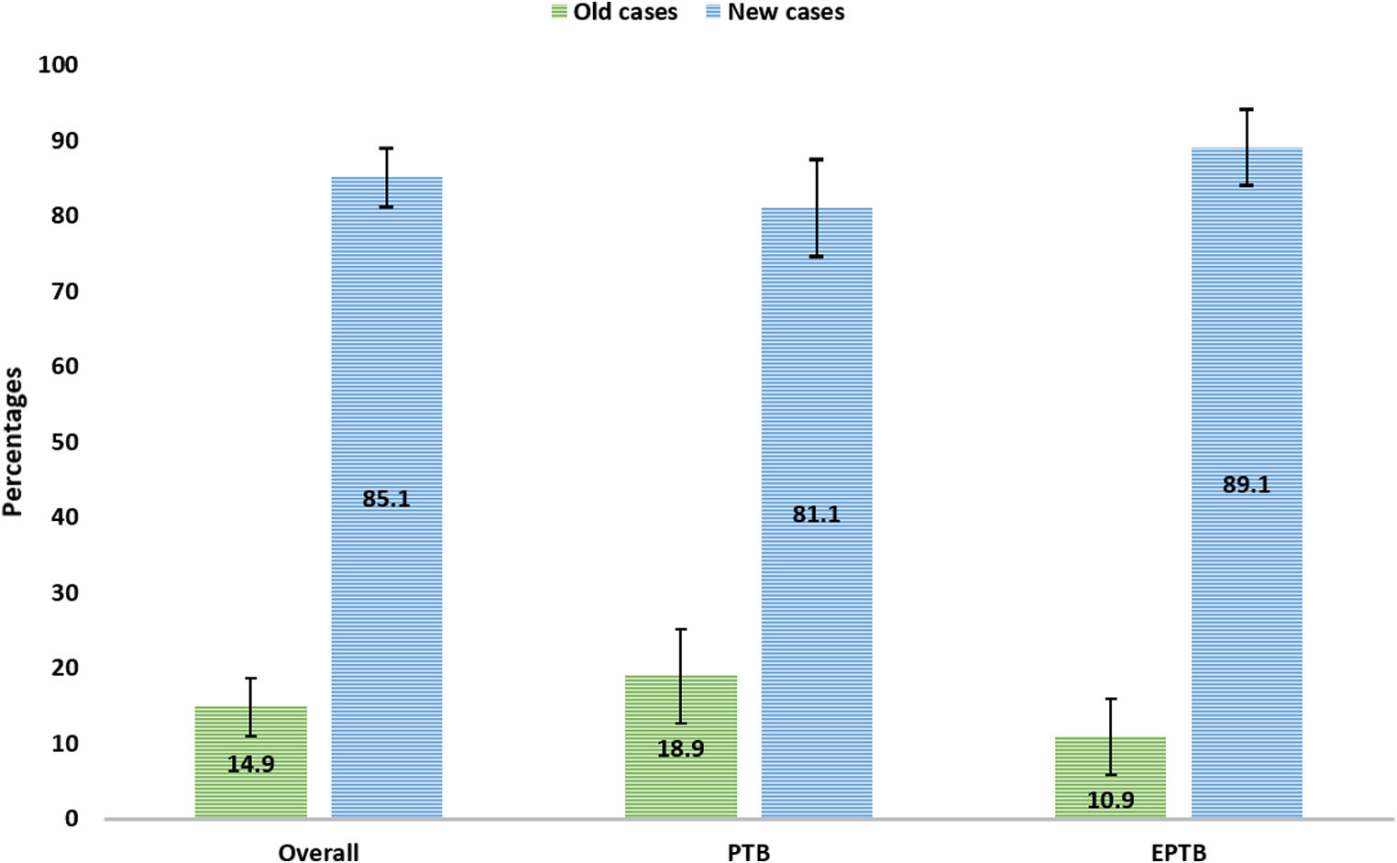
Proportion of old and new cases of T2D among the patients with pulmonary (PTB) and extrapulmonary tuberculosis (EPTB)

**FIGURE 2:**
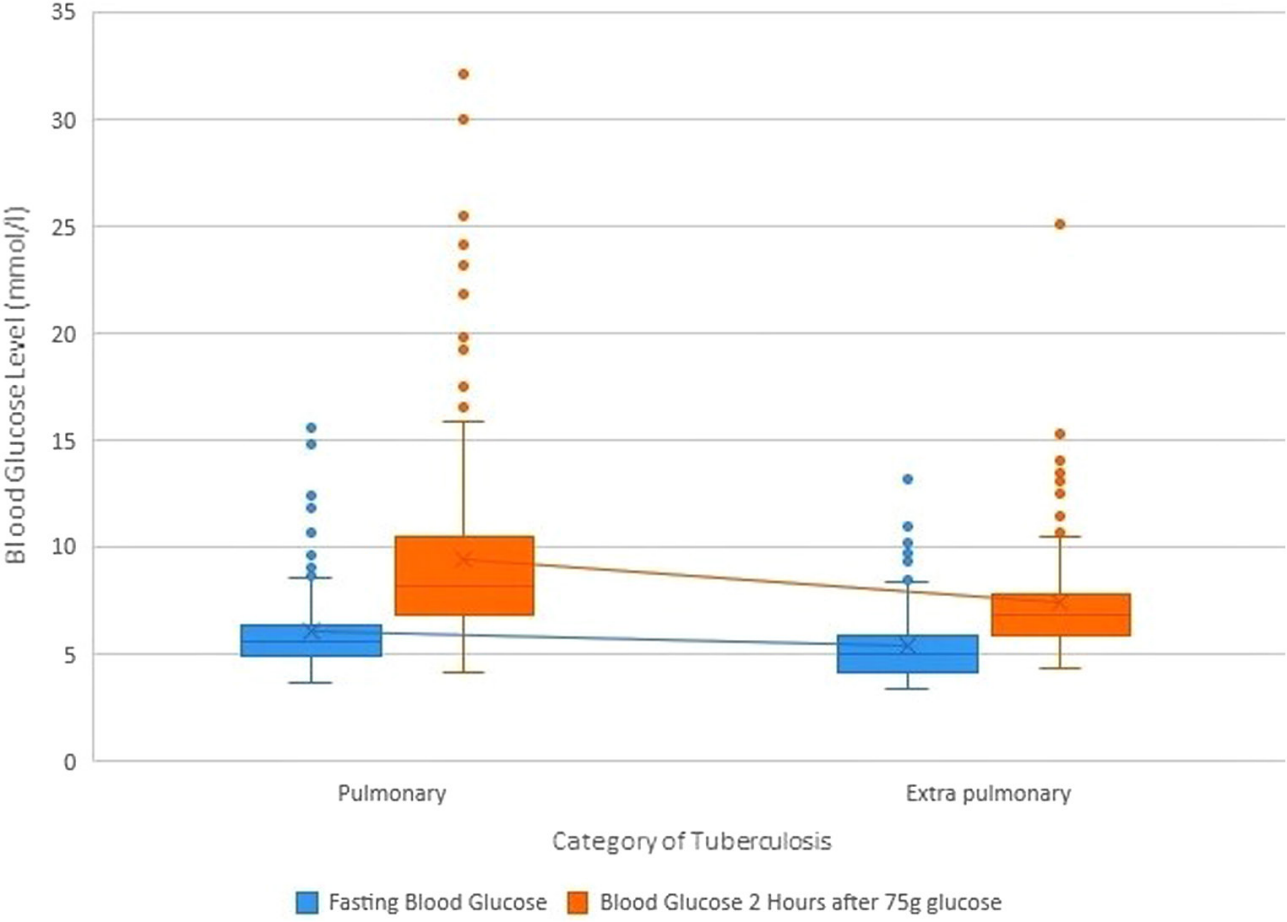
Blood glucose levels according to the types of tuberculosis

Table 2 depicted the blood glucose level, T2D and pre‐DM among the tuberculosis patients. The overall prevalence of T2D among the study participants was 19.1% (CI: 15.5–23.9). The proportion of new and old cases was 85.1% and 14.9%, respectively (Figure 1). Group‐wise distribution highlighted a higher proportion of DM (*p* = .002) in the PTB group (26.3% CI: 19.8–32.8) than in the EPTB group (13.1%, CI: 8.1–18.1), although more risk factors were common in the EPTB group. However, the new cases were slightly higher in the EPTB group (89.1%) compared with the PTB group (81.1%) (Figure 1). The proportion of pre‐DM was also higher in the PTB group (34.3%, CI: 27.3–41.3). The proportion of IFG was low in both groups, but a high trend of IGT was observed across the groups, with a higher proportion in the PTB group (35.4%, CI: 28.3–42.5). Comparative analysis also showed a higher FPG (6.1 ± 2.2 mmol/L) and 2 hours PG (9.5 ± 4.4 mmol/L) in the PTB group (Figure 2).

**TABLE 2:** Blood glucose level, diabetes and pre‐diabetes among the tuberculosis patients, *n* = 350

Variables	Total (*n* = 350)		Pulmonary TB (*n* = 175)		Extrapulmonary TB (*n* = 175)		*p*‐value*
	*n* (%)	95% CI	*n* (%)	95% CI	*n* (%)	95% CI	
Fasting plasma glucose (FPG)^†^	5.7 (2)		6.1 (2.2)		5.4 (1.6)		<.001
2 h plasma glucose (2‐h PG)^†^	8.4 (3.7)		9.5 (4.4)		7.4 (2.5)		<.001
Diabetes	69 (19.7)	15.5–23.9	46 (26.3)	19.8–32.8	23 (13.1)	8.1–18.1	.002
Pre‐diabetes	86 (24.6)	20.1–29.1	60 (34.3)	27.3–41.3	26 (14.9)	9.6–20.2	<.001
Impaired fasting glucose	7 (2)	0.5–3.5	3 (1.7)	−0.2 to 3.6	4 (2.3)	0.1–4.5	.70
Impaired glucose tolerance	89 (25.4)	20.8–30	62 (35.4)	28.3–42.5	27 (15.4)	10.1–20.7	.0001

Abbreviation: TB, tuberculosis.

* Statistically significant at the level of *p* < .05.

^†^ Representing mean and standard deviation in mmol/l; *p*‐value is calculated by Mann–Whitney *U* test.

### 3.3 Association of T2D and pre‐DM with type of tuberculosis

In Table 3, after adjusting for modifiable risk factors (tobacco consumption, overweight and central obesity) of high blood glucose (pre‐DM, T2D), a highly significant association was elucidated between blood glucose status and the type of TB. We found PTB as the predictor of both pre‐DM (AOR: 4.488; CI: 2.531–7.958; *p* < .001) and T2D (AOR: 4.280; CI: 2.305–7.946; *p* < .001).

**TABLE 3:** Association between high blood glucose (diabetes/pre‐diabetes) and type of tuberculosis among the study population using multinomial logistic regression analysis where ‘normal blood glucose level’ was considered as reference for the dependent variable (*n* = 350)

High blood glucose	Type of TB	UAOR	*p*‐value*	AOR^b^	95% Confidence Interval for odds ratio	
					Lower Bound	Upper Bound
Diabetes	PTB	1.454	<.001	4.280	2.305	7.946
	EPTB (Ref.)	0^a^				
Pre‐diabetes	PTB	1.501	<.001	4.488	2.531	7.958
	EPTB (Ref.)	0^a^				

Abbreviations: AOR, adjusted odds ratio; EPTB, extrapulmonary tuberculosis; PTB, pulmonary tuberculosis; Ref, reference; TB, tuberculosis; UAOR, unadjusted odds ratio.

^a^ This parameter is set to zero (0) because it is redundant

^b^ Adjusted for modifiable risk factors of high blood glucose: current tobacco use, overweight and central obesity.

* Statistically significant at the threshold of *p* < .05.

## 4 DISCUSSION

Diabetes and pre‐DM were highly prevalent among TB patients in the tertiary level specialized hospitals in Bangladesh. Particularly, people with PTB had a higher prevalence of DM or pre‐DM. The presence of risk factors for T2D in the subgroups of TB varied. While hypertension and central obesity were more common in people with EPTB, tobacco consumption was more common in people with PTB. However, almost one in five patients of both subgroups of TB had a family history of DM. PTB was detected as the statistically significant predictor of both pre‐DM and T2D.

To our knowledge, this was the first study in Bangladesh that aimed to identify the burden of diabetes or hyperglycaemia in subgroups of TB patients. The study showed that people with PTB were more affected by T2D or hyperglycaemia. The result was consistent with other epidemiological studies conducted in India,^25^, ^26^, ^27^ Mexico^28^ and Vietnam.^29^ In these studies, the prevalence of diabetic patients with PTB and EPTB ranges from 15% to 35% and 8% to 26%, respectively.^25^, ^26^, ^27^, ^28^, ^29^ A cross‐sectional study conducted in Bangladesh also identified that PTB patients made eighty‐five per cent of the burden of diabetes in the TB population.^14^ However, it was uncertain why people with PTB faced a high prevalence of T2D. One possibility was a higher prevalence of tobacco consumption in the PTB group could influence the burden of T2D. Studies demonstrated diabetic smokers had a fivefold increased risk of a pretreatment positive smear than did non‐diabetic non‐smokers.^30^ Although studies reported production of high free radicals in smokers contributed to the development of T2DM,^31^, ^32^ no studies had found that investigated the influence of tobacco consumption on T2D in association with PTB patients.

In the current study, the overall prevalence of diabetes in patients with TB was higher than the global pooled prevalence (15.3% 95% CI 14.1–16.6) of diabetes in patients with TB but similar to the Southeast Asia (19.0% 95% CI 16.2–32.5).^33^ Southeast Asia experiences the highest prevalence of diabetes in TB patients. Within Southeast Asia, the highest prevalence was reported in Sri Lanka (24.1 95% CI 16.6–32.5), and then in India (19.9 95% CI 16.8–23.2).^33^ In Bangladesh, the pooled prevalence was reported as 10.6% (95% CI 7.2–14.5), which is close to the reported prevalence for the IDF regions that experienced the least prevalence of diabetes in TB patients that includes Africa (8·0% 95% CI 5·9–10·4), south and central America (7·7% 95% CI 6·9–8·6), and Europe (7·5% 95% CI 5·2–10·2).^33^ Contradictory to the previous literature, the current study showed a higher proportion of diabetes, which is consistent with the prevalence of diabetes in TB patients in Southeast Asia.^14^, ^34^, ^35^

The clinical characteristics of the study population might influence the higher proportion of T2D in TB patients in the current study compared to previously published studies. In the previous studies, the participants were recruited from the community and local hospitals, whereas in this study, the participants were from tertiary level tuberculosis hospitals. In these hospitals, people with TB refer from primary or secondary facilities if the patients need advanced diagnostic and treatment facilities. Particularly suspected multidrug‐resistant tuberculosis (MDR‐TB) patients referred from other parts of the country. The presence of MDR‐TB patients among the study population might contribute to a high proportion of DM in TB patients. A previous study of Bangladesh reported that MDR‐TB patients had more T2D (13.6%) than drug‐sensitive TB patients (6.5%).^35^ This finding indicates that patients with TB in tertiary level hospitals in Bangladesh may have a higher prevalence of T2D.

The current study found a highly significant association between PTB and high blood glucose (T2D, AOR: 4.280, *p* < .001; pre‐DM, AOR: 4.488, *p* < .001) that first time evaluated among Bangladeshi TB patients. Globally, very few studies tried to assess whether TB increases the risk of diabetes. It is difficult to reach a valid conclusion that TB is a risk factor for diabetes mellitus. However, our current findings coincided with previous studies that reported either hyperglycaemia or diabetes among TB patients.^36^, ^37^, ^38^ A study reported glucose intolerance among 10.4% of the participants and diabetes in 8.6% without DM. It also showed the increased prevalence of DM up to 17.4% compared with a matched control group of community‐acquired pneumonia.^37^ Similarly, another study found abnormal results for 42.6% of the participants, of whom 5.6% and 37.0% had diabetes and IGT, respectively.^36^ The proposed underlying pathophysiological mechanism of TB‐induced DM is TB pancreatitis as well as pancreatic endocrine hypofunction which may lead to IGT or new‐onset diabetes.^39^, ^40^

The strengths of this study include (i) inclusion of two common clinical types of TB as the study population that rarely assessed and compared for pre‐DM and T2D, (ii) use of OGTT to diagnose new cases of T2D and (iii) recruitment of study population from two national institutes dedicated to TB treatment in Bangladesh and patients from all over the country visited these institutions for better care. Again, this study provided the first comparative data of pre‐DM and T2D among the patients with PTB and EPTB currently lacking in Bangladesh. In a low‐resource setting, it will guide to target the patients with TB who are at risk for diabetes.

This study had several limitations. First, the participants selected purposively might elevate the risk of selection bias. Second, a few self‐reported risk factors were reported that might be associated with recalled bias. Finally, only two study sites limited the generalizability of findings as TB care is not similar all over the country, and disparity exists.

## 5 CONCLUSION

In conclusion, having a high proportion of DM and pre‐DM among the TB population highlighted the need for DM screening and regular glycaemic level monitoring along with TB treatment. Screening and monitoring DM may help in detecting undiagnosed DM and having favourable TB treatment outcomes by controlling blood glucose levels. Identification of the subgroups of TB patients who are more affected by diabetes or pre‐DM in this study have helped in targeting the diabetes screening intervention in TB patient. While both PTB and EPTB patients may have the benefit of screening and monitoring, particularly people with PTB may get the more benefit of the intervention as the proportion of DM is significantly higher in this population and a notable association has been observed. Current national TB treatment guidelines should also emphasize regular screening and monitoring of DM as a part of the standard of care.

## CONFLICT OF INTEREST

The authors declare no conflict of interest.

## AUTHOR CONTRIBUTIONS

**Afsana Habib Sheuly:** Conceptualization (lead); Funding acquisition (lead); Investigation (lead); Methodology (lead); Project administration (lead); Writing – review & editing (supporting). **S. M. Zahid Hassan Arefin:** Conceptualization (supporting); Funding acquisition (supporting); Investigation (supporting); Methodology (supporting); Project administration (supporting); Writing – review & editing (supporting). **Lingkan Barua:** Data curation (lead); Formal analysis (lead); Methodology (supporting); Software (supporting); Writing – original draft (lead); Writing – review & editing (equal). **Muhammed Shahriar Zaman:** Data curation (equal); Formal analysis (equal); Methodology (supporting); Software (supporting); Writing – original draft (equal); Writing – review & editing (supporting). **Hasina Akhter Chowdhury:** Conceptualization (equal); Data curation (equal); Formal analysis (equal); Investigation (equal); Methodology (equal); Project administration (equal); Supervision (lead); Writing – original draft (supporting); Writing – review & editing (supporting).

## ETHICAL APPROVAL

Approval of the research protocol: The study was approved by the Ethical Review Committee of Bangladesh University of Health Sciences and conducted in accordance with the Declaration of Helsinki (October 2013).

Informed Consent: All subjects gave their written informed consent for inclusion before they participated in the study.

Approval date of Registry and the Registration No. of the study: BUHS‐ERC/EC/15/007 on 8 January 2015.

Animal Studies: N/A.

## Supporting information

Table S1

## Data Availability

All data relevant to the study are included in the article.
