# Supplementary material for: Prevalence of type 2 diabetes and pre‐diabetes among pulmonary and extrapulmonary tuberculosis patients of Bangladesh: A cross‐sectional study
Source: Endocrinol Diabetes Metab. 2022 Mar 8;5(3):e00334. doi: 10.1002/edm2.334 (PMC9094490; doi:10.1002/edm2.334)
Supplement: Supplementary file 1 — Table S1 [file EDM2-5-e00334-s001.pdf]

Table S1: **Sample size calculation**	
**For prevalence of type 2 diabetes among tuberculosis (TB) patients**	**For comparing proportion of pulmonary TB and extrapulmonary TB**
For prevalence of type 2 diabetes mellitus among TB patients sample size was calculated using the formula: n = z2q/r2p<br>Where z=1.96 (95% confidence interval)<br>p=prevalence of diabetes among tuberculosis patient: 25.3%[1]<br>q= (100-25.3) % =74.7%<br>r= relative error 25% of prevalence<br>Sample size (n) was 290<br><br>Reference 1: Baker MA, Harries AD, Jeon CY, et al. The impact of diabetes on tuberculosis treatment outcomes: A systematic review. BMC Medicine. 2011; 9.	For comparison among pulmonary and extra- pulmonary patients sample size calculation was done by<br>n= {z1-α/2√[2p(1-p)]+z1-β√[p1(1-p1)+p2(1-p2)]}²/(p1-p2)²<br>Where,<br>Prevalence of diabetes in pulmonary TB patients (p1) =27.2%[2]<br>Prevalence of diabetes in extra pulmonary TB patients (p2) =14.8%2<br>z = 1.96 (95% confidence interval)<br>Power 80%<br>The sample size was 104 in each pulmonary and extra-pulmonary group.<br>Reference 2: Jeon CY, Harries AD, Baker MA, et al. Bi-directional screening for tuberculosis and diabetes: a systematic review. Tropical Medicine & International Health. 2010; 15:1300-1314.
Finally, the sample size was 175 for each pulmonary TB and extra pulmonary TB for the sub population – male /female and, hospital registered inpatient and DOTs registered. Final sample size was arrived at using adjustment for 10% nonresponse rate.	
